# Mechanisms of Age-Dependent Loss of Dietary Restriction Protective Effects in Acute Kidney Injury

**DOI:** 10.3390/cells7100178

**Published:** 2018-10-22

**Authors:** Nadezda V. Andrianova, Stanislovas S. Jankauskas, Ljubava D. Zorova, Irina B. Pevzner, Vasily A. Popkov, Denis N. Silachev, Egor Y. Plotnikov, Dmitry B. Zorov

**Affiliations:** 1 Faculty of Bioengineering and Bioinformatics, Lomonosov Moscow State University, 119992 Moscow, Russia; andnadya12@yandex.ru (N.V.A.); popkov.vas@gmail.com (V.A.P.); 2 A.N. Belozersky Institute of Physico-Chemical Biology, Lomonosov Moscow State University, 119992 Moscow, Russia; jankauskas.ss@gmail.com (S.S.J.); lju_2003@list.ru (L.D.Z.); irinapevzner@mail.ru (I.B.P.); silachevdn@belozersky.msu.ru (D.N.S.)

**Keywords:** aging, dietary restriction, acute kidney injury, mitochondria, autophagy, mitophagy, ischemia, renal tubular cells

## Abstract

Dietary restriction (DR) is one of the most efficient approaches ameliorating the severity of different pathological conditions including aging. We investigated the protective potential of short-term DR in the model of acute kidney injury (AKI) in young and old rats. In kidney tissue, the levels of autophagy and mitophagy were examined, and proliferative properties of renal cells obtained from rats of different age were compared. DR afforded a significant nephroprotection to ischemic kidneys of young rats. However, in old rats, DR did not provide such beneficial effect. On the assessment of the autophagy marker, the LC3 II/LC3 I ratio, and after staining the tissue with LysoTracker Green, we concluded that in old rats activity of the autophagic-lysosomal system decreased. Mitophagy, as assessed by the levels of PINK-1, was also deteriorated in old animals. Renal cells from old rats showed impaired proliferative capacity, a worse rate of recovery after ischemic injury, increased levels of oxidative stress, accumulation of lipofuscin granules and lower mitochondria membrane potential. The results suggest that the loss of DR benefits in old animals could be due to deterioration in the autophagy/mitophagy flux.

## 1. Introduction

Caloric or dietary restriction (DR), defined as a reduced food intake without evident signs of malnutrition, is an approach which for many organisms has shown an increase in lifespan and a slowdown in the manifestation of age-associated diseases [1]. DR improves the functioning of organs and tissues including those of old animals. For instance, even a short-term DR postpones the onset of age-dependent changes in the heart, brain, liver, and kidneys [2,3,4,5]. Moreover, DR affords increased tolerance to such harmful challenges as myocardial infarction [6], brain diseases [7], hypertension [8], and diabetes mellitus [9]. Recently, several studies have been initiated to verify the beneficial effects of DR on longevity and the attenuation of pathologies in humans [10,11].

A broad study of the mechanisms underlying the beneficial effects of DR pointed to autophagy as a key element of the protective mechanism [12]. In particular, due to the activation of autophagy mechanisms, one can expect a decrease in the levels of damaged macromolecules and organelles, for example, mitochondria. However, with age, the processes of autophagy gradually deteriorate, which leads to a disruption in the quality control of macromolecules and organelles [13]. DR at least partially can restore the ability of the aging organism’s cells to eliminate damaged components and to improve tolerance to various damaging factors, which results in the “rejuvenation” of tissues [1].

Even though the protective effect of DR has been revealed quite a long time ago and has been intensively studying [1,14], there is too little data covering its beneficial effects in the kidney tissue. Nevertheless, previous studies have shown that DR exhibits a protective effect in acute cisplatin and cadmium nephrotoxicity [15,16,17], as well as in the model of chronic kidney disease and diabetic nephropathy [18,19], i.e., DR affects both acute and chronic kidney injuries.

Acute kidney injury (AKI) is a widespread pathology affecting more than 20% of hospitalized patients in developed countries [20]. Approximately 90% of patients with this disease are individuals of 64+ years of age [21]. Since the majority of patients with AKI are elderly, it is essential to study the tolerance of the kidneys specifically in old organisms. It should be considered that with age, the kidney tissue becomes more sensitive to various damaging factors due to progressive molecular, structural and functional deleterious rearrangements [22].

Since available methods for the treatment of AKI are scarce and applied strategies are mostly symptomatic and mostly limited by the use of hemodialysis, the development of alternative specific approaches for therapy is needed [23]. However, the effectiveness of these elaborated approaches should be tested both in young and old animals, since approaches that have been shown to be efficient in young organisms, not always retained their protective effects in the elderly [24]. In particular, ischemic pre- and postconditioning, demonstrating a significant reduction of the severity of ischemic injury in the renal tissue of young animals, had no effect in old animals [25,26].

Since the proven ability of DR to reduce the severity of AKI after ischemia/reperfusion (I/R) has been obtained in rodents of a young age [27,28,29], the goal of our study was to compare the therapeutic potential of DR in animals of different age and to assess the mechanisms of ischemic tolerance underlying the beneficial effects of DR, particularly the autophagic pathway.

## 2. Materials and Methods

### 2.1. Animals

Experiments were performed on male outbred rats of different ages: young (3–4 month old, 300–400 g), adult (12 months old, 500–600 g) and old rats (20–23 months old, 550–700 g). The animal protocols were evaluated and approved by the animal ethics committee of A.N. Belozersky Institute of Physico-Chemical Biology (Protocol 2/13 from 8 April 2013). They were in accordance with the Federation of Laboratory Animal Science Associations (FELASA) guidelines.

### 2.2. Dietary Protocol

The amount of food consumed ad libitum (AL) was approximately 25 g/day for young rats and 30–33 g/day for adult and old rats, as measured by weighing the remaining food for two weeks. DR was performed for 4 weeks by limiting the amount of food by 35% (35% DR) or by 25% (25% DR) of the daily intake. Food was administered once daily at 2:00 pm. Fasting (100% DR) lasted for 3 days without any access to food. After kidney I/R, ad libitum administration of food was restored. For all groups, the free access to water was implemented.

### 2.3. Kidney I/R Protocol

Rats were anesthetized with chloralhydrate (300 mg/kg, i.p.) and subjected to 40-min warm ischemia of the left kidney as previously described [30]. Briefly, the renal vascular bundle was occluded with a microvascular clip for 40 min. Circulation was restored by removing the clip. The lack of blood flow during ischemia and its restoration during reperfusion were assessed visually. Nephrectomy of the right kidney was performed simultaneously with the ischemia of the left one. During operation, the body temperature of the rats maintained at 37 ± 0.5 °C. Blood samples were taken 48 h after I/R from the carotid artery to determine the blood urea nitrogen (BUN) and serum creatinine (SCr) using AU480 Chemistry System (Beckman Coulter, Brea, CA, USA).

### 2.4. Western Blotting

Rats were sacrificed and kidneys were taken 48 h after I/R. Kidney tissue was homogenized with a glass-Teflon homogenizer in a PBS buffer, containing 10 mM phenylmethylsulfonylfluoride at 4 °C. Kidney samples were loaded onto 15% Tris–glycine polyacrylamide gels (10 µg protein/lane). After electrophoresis, gels were blotted onto PVDF membranes (Sigma-Aldrich, St. Louis, MO, USA). Membranes were blocked with 5% non-fat milk in PBS with 0.05% Tween-20 and subsequently incubated with primary antibodies: anti-LC3 A/B monoclonal rabbit 1:1000 (Cell Signaling, Danvers, MA, USA), anti-ubiquitin polyclonal rabbit 1:1000 (Abcam, Cambridge, MA, USA), anti-PINK1 polyclonal rabbit 1:1000 (Abcam, Cambridge, MA, USA), anti-SIRT-3 monoclonal rabbit 1:2000 (Cell Signaling, Danvers, MA, USA), anti-β-actin monoclonal mouse 1:1500 (Sigma-Aldrich, St. Louis, MO, USA), and anti-COX IV monoclonal mouse 1:1500 (Abcam, Cambridge, MA, USA). Membranes were stained with secondary antibodies: anti-rabbit IgG or anti-mouse IgG conjugated with horseradish peroxidase 1:7500 (Jackson ImmunoResearch, Ely, UKUSA). Detection was performed by a Chemidoc MP (BioRad, Hercules, CA, USA). Protein concentration was measured by bicinchoninic acid assay (Sigma-Aldrich, St. Louis, MO, USA).

Since on western blots LC3 is represented by several bands, some of the young rats were subjected to chloroquine (20 mg/kg/day, i.p.) for 3 days to localize the position of the LC3 II band, which increases after chloroquine inhibition of its lysosomal degradation.

The rat kidney mitochondria for western blotting were isolated by differential centrifugation in the medium containing 0.25 M sucrose, 10 mM HEPES, 1 mM EDTA, 0.1% BSA, with a pH 7.4.

### 2.5. Lysosomes Imaging

Kidneys were excised 24 h after I/R and placed in the incubation medium (DMEM/F12 without sodium bicarbonate) to wash the blood out. Then, 100–150 µm thick sections through the cortical zone of the kidneys were made. Vital tissue slices were washed using the incubation medium (all procedures and incubation were done at 25 °C) and loaded for 30 min with 1 µM LysoTracker Green (Invitrogen, Thermo Fisher Scientific, Waltham, MA, USA). Kidney slices were imaged with an LSM510 inverted confocal microscope (Carl Zeiss, Jena, Germany). Images were processed using ImageJ software (NIH, Bethesda, MD, USA).

### 2.6. Primary Culture of Renal Tubular Cells (RTCs)

Kidneys from young and old intact rats were excised under aseptic conditions. The cortex of the kidneys was ground, carefully washed from blood, and dissociated by type II collagenase (0.25%, 30 min at 37 °C; Gibco, Thermo Fisher Scientific, Waltham, MA, USA). Pieces of tissue were removed, and the suspension was centrifuged for 5 min at 100× *g* after which the renal tubules were pelleted and the dissociated cells remaining in the suspension were discarded. The pellet was resuspended in a complete culture medium (DMEM/F12 with 10% FCS and 10 ng/mL EGF (Invitrogen, USA)) and seeded onto 6-well plates, or special iCelligence 8-well plates, or 35-mm glass-bottom Petri dishes (FluoroDish, WPI, Sarasota, Florida, USA). Cultures were kept at 37 °C under 5% CO_2_ in a humidified atmosphere. The culture medium was changed after 48 h in order to eliminate non-adherent cells and residual cellular fragments. RTCs formed confluent monolayers 5 days after plating, therefore, all procedures were carried out on 2–4 days in vitro.

Cultures of RTCs were loaded with 1 µM MitoTracker Red (Invitrogen, Thermo Fisher Scientific, Waltham, MA, USA). Incubation was conducted in DMEM/F12 without sodium bicarbonate for 30 min at 37 °C. Then, RTCs were washed with saline and dissociated with 0.05% Trypsin-EDTA. Cells were resuspended in 1.5 mL DMEM/F12. The fluorescence intensity was evaluated by flow cytometry as described below.

To evaluate the autophagy activation, RTCs were incubated with 500 nM rapamycin (Sigma-Aldrich, St. Louis, MO, USA) and 30 μM chloroquine (Sigma-Aldrich, St. Louis, MO, USA). Rapamycin and chloroquine were dissolved in a complete medium (DMEM/F12 with 10% FCS). Incubation was performed for 3 h, after which RTCs were washed with saline and loaded with 2 µM Cyto-ID (Enzo Life Sciences, New York, NY, USA). Further steps were the same as for MitoTracker Red staining.

### 2.7. Non-Cultured Renal Tubular Cells (NC-RTCs) from the Kidneys of Young and Old Rats

In addition to primary cell cultures of RTCs, we also used NC-RTCs, which were in situ isolated from kidneys of young and old rats. Cell suspensions were analyzed in situ without any growth in the culture medium, i.e., immediately after isolation.

Similar to RTCs preparation, pieces of cortex were ground and dissociated by type II collagenase (0.25%, 30 min at 37 °C). The pieces of tissue were repeatedly resuspended and kept for 2 min to pellet large pieces. The supernatant was centrifuged for 5 min at 100× *g* after which the renal tubules were precipitated. The supernatant was discarded and tubules were again incubated with type II collagenase (0.25%, 10 min at 37 °C) one more time, after which the suspension was intensively pipetted. Then, the suspension was centrifuged for 5 min at 100× *g* for sedimentation of undissociated tubules, the resulting supernatant was centrifuged for 5 min at 600× *g* to pellet the cells. The resulted suspensions of NC-RTCs were diluted to concentration 1 × 10^6^ cells/mL and incubated for 30 min with 1 μM MitoTracker Green (Invitrogen, Thermo Fisher Scientific, Waltham, MA, USA), 1 μM MitoTracker Red (Invitrogen, Thermo Fisher Scientific, Waltham, MA, USA), or 10 μM DCF-H2 (Invitrogen, Thermo Fisher Scientific, Waltham, MA, USA) at 37 °C. The fluorescence intensity in cells was analyzed by flow cytometry.

### 2.8. Flow Cytometry

Flow cytometry was performed by using Cytomics FC500 (Beckman Coulter, Brea, CA, USA). Primary cultures of RTCs and NC-RTCs were obtained and stained as described above. Cyto-ID, MitoTracker Green and DCF-mediated fluorescence were measured on the FL1 channel, while MitoTracker Red-mediated fluorescence was evaluated on the FL3 channel. Argon laser with λ_ex_ = 488 nm was used to excite the fluorescence.

### 2.9. Real-Time Cell Proliferation Monitoring

Analysis of cells growth kinetics was performed using an RTCA iCELLigence™ instrument (ACEA, San Diego, CA, USA). This method is based on using electrical impedance of cell-covered electrodes [31] and may be used in particular for studies of RTCs proliferation and death [32]. The iCELLigence RTCA instrument was placed in a humidified incubator at 37 °C and 5% CO_2_. RTCs from young and old rats were seeded on 8-well plates with microelectrodes; 48 h after, the medium was changed to dispose of unattached and dead cells. After the next 24 h, cells were subjected to 17 h of oxygen-glucose deprivation (OGD): the medium was changed to DPBS (Dulbecco’s phosphate-buffered saline) and oxygen was replaced with N_2_ in multi-gas incubator Galaxy 170R (Eppendorf/NewBrunswick, Hamburg, Germany). After OGD, the DPBS was changed back to the culture medium with a normoxic O_2_ content. Proliferation rates were detected during 48 h after OGD.

### 2.10. Lipofuscin Detection

RTCs from young and old rats were obtained and cultured as described above. A neonatal culture of RTCs was prepared from kidneys of 7-days-old rats by the same protocol as for young and old rats, but with a less prolonged incubation with collagenase type II (0.125%, 15 min at 37 °C). On the 2nd day in vitro, cells were washed with saline and fixed in a 10% neutral buffered formalin solution for 15 min, washed out, and lipofuscin red autofluorescence was analyzed by an LSM510 inverted confocal microscope (Carl Zeiss, Jena, Germany). Analysis of fluorescence was performed in glass-bottom dishes with the excitation at 488 nm and 543 nm and emission collected beyond 585 nm.

Lipofuscin red autofluorescence was also measured by flow cytometry using Cytomics FC500 (Beckman Coulter, Brea, CA, USA). On the 3rd day in vitro, RTCs were washed, dissociated with 0.05% Trypsin-EDTA and suspension was centrifuged for 5 min at 600× *g*. The pellet was resuspended in 2 mL of DMEM/F12 without sodium bicarbonate. The autofluorescence of unstained RTCs was detected on FL5 channel with λ_ex_ = 635 nm.

### 2.11. Statistics

Values are presented as mean ± SEM. Comparisons between groups were made using the Mann–Whitney U-test. Data were analyzed in Microsoft Excel.

## 3. Results

### 3.1. Effect of DR on the Severity of AKI in Young and Old Rats

**Figure 1:**
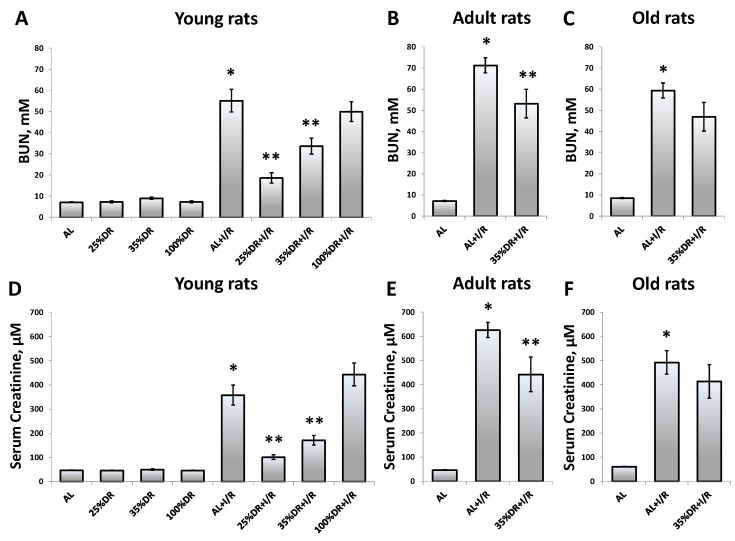
The evaluation of kidney function by the blood urea nitrogen (BUN) and serum creatinine (SCr) in young, adult, and old rats kept on an ad libitum (AL) diet or using different protocols of dietary restriction (DR). (**A**) Assessment of renal function in young (3–4 months) control days (100% DR). Evaluation of the severity of acute kidney injury (AKI) in these groups of rats 48 hours after I/R; (**B**) Assessment of kidney function and severity of AKI 48 hours after I/R in adult (12 months) rats; (**C**) Assessment of kidney function and severity of AKI 48 hours after I/R in old (20–23 months) rats; (**D**–**F**) Evaluation of SCr concentration in young, adult, and old rats on AL or DR 48 hours after I/R. Control groups contained more than 3 rats, while groups with I/R included more than 6 rats. * *p* < 0.05 compared to AL, ** *p* < 0.05 compared to AL + I/R.

Since ischemic injury is the most common factor leading to AKI [23], we used kidney I/R as a model, evaluating AKI severity by BUN and SCr measuring. There was about an 8-fold increase of BUN 48 h after I/R in young AL rats (55.1 mM ± 5.4 mM of BUN vs. 7.1 mM ± 0.2 mM in intact young rats) (Figure 1A). In adult and old rats, BUN increased up to 71.3 mM ± 3.6 mM and 59.4 mM ± 6.9 mM, correspondingly (Figure 1B,C).

In young rats, we tried different protocols of DR; 25% DR and 35% DR significantly reduced BUN 48 h after I/R compared to the AL group. However, 35% DR decreased BUN twice more effective than 25% DR which resulted in a drop of BUN by 39% against the AL group (33.6 mM ± 3.8 mM and 55.1 mM ± 5.4 mM, respectively), whereas 35% DR reduced BUN to 18.6 mM ± 2.4 mM compared to 55.1 mM ± 5.4 mM in the AL group. Fasting for 3 days (100% DR) did not cause a significant change in the severity of AKI in young rats.

In adult and old rats, we only tested 35% DR since it showed a higher efficiency in young rats. Some decrease of the BUN level in the 35% DR group was found in adult rats (aged 12 months) 48 h after I/R: 53.2 mM ± 6.7 mM as compared to 71.3 mM ± 3.6 mM in the AL group (Figure 1B). In contrast to young and adult animals, in old rats, 35% DR did not result in any significant nephroprotective effect (Figure 1C). Of note, no morbidity or mortality was observed as a result of different DR protocols.

Changes in renal functions were proven by detecting SCr concentrations. We evaluated SCr levels in all experimental groups of rats and observed the same tendencies as when we measured the BUN (Figure 1D–F).

### 3.2. Autophagy in Renal Tissue of Young and Old Rats

**Figure 2:**
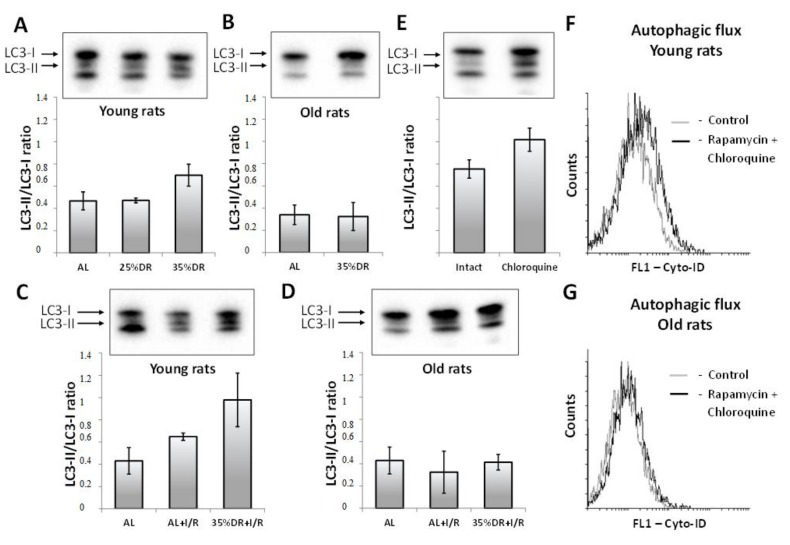
The assessment of autophagic activity in young and old rats. (**A**,**B**) LC3 II/LC3 I ratio in kidney homogenates of young and old control rats on AL or DR; (**C**,**D**) LC3 II/LC3 I ratio in kidney homogenates of young and old rats on AL or DR 48 hours after I/R; (**E**) LC3 II/LC3 I ratio in kidney homogenates of young rats after the injections of chloroquine (20 mg/kg/day) for 3 days; (**F**,**G**) Autophagic flux in primary cultures of renal tubular cells (RTCs) obtained from the kidneys of young and old rats, after incubation with rapamycin and chloroquine.

It is well known that DR is a strong factor that stimulates the processes of autophagy [33]. Indeed, we found an increase in the LC3 II/LC3 I ratio in the kidneys of young rats after 4 weeks of 35% DR. On the other hand, 25% DR did not result in a significant increase of the LC3 II/LC3 I ratio (Figure 2A). In contrast to the young rats, in old rats, 35% DR did not cause an augmentation of the LC3 II/LC3 I ratio compared to the AL old rats (Figure 2B).

I/R is also able to activate autophagic signaling pathways [34]. In the kidneys of young rats taken 48 h after I/R, we observed an increased LC3 II/LC3 I ratio (Figure 2C). The ratio increased even more in the group of rats that underwent 35% DR before exposure to I/R (Figure 2C). In old rats, such changes of LC3 II/LC3 I were not revealed: in these animals, the ratio remained the same in all groups (Figure 2D).

Since on western blots LC3 is represented by several bands, some of the young rats were subjected to chloroquine for 3 days to localize the position of the LC3 II band. A relatively low concentration of chloroquine was chosen because higher doses of chloroquine caused a decrease in rats’ food intake. The band that appeared after chloroquine treatment was assumed as LC3 II (Figure 2E).

A stronger activation of autophagy in young rats than in old ones was confirmed by examination of primary RTCs cultures with Cyto-ID which is selectively accumulated in autophagosomes and autophagolysosomes [35]. The aggregate effect of the autophagy activator, rapamycin, and the inhibitor of the late stages of autophagy, chloroquine, resulted in a great increase in the fluorescence intensity of Cyto-ID in RTCs from young rats (Figure 2F). RTCs from old rats did not show any increase in Cyto-ID fluorescence in response to rapamycin and chloroquine (Figure 2G). Moreover, RTCs from old rats at the baseline showed a weaker intensity of Cyto-ID fluorescence than RTCs from young rats (Figure 2F,G).

**Figure 3:**
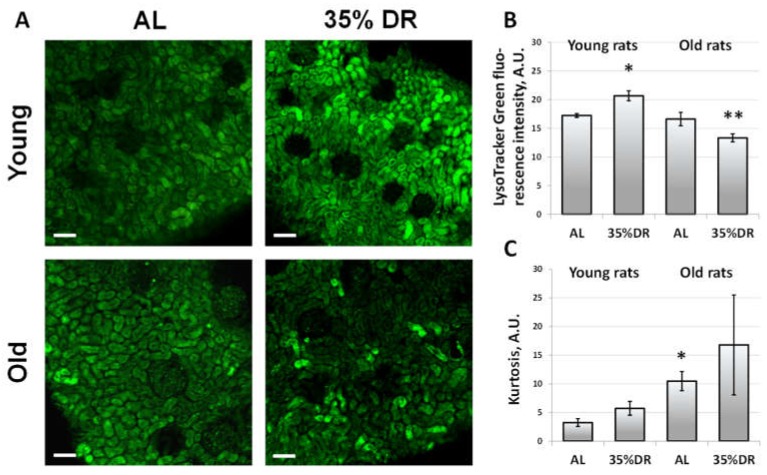
The staining of vital renal cortex slices of young and old rats fed AL or subjected to 35% DR with LysoTracker Green. (**A**) Confocal microscopy after LysoTracker Green staining. The scale bar indicates 100 µm. (**B**) Quantification of LysoTracker Green fluorescence intensity on the vital renal slices of young and old rats; (**C**) Quantification of the coefficient of kurtosis of LysoTracker Green distribution on the vital renal slices of young and old rats. * *p* < 0.05 compared to the AL group of young rats, ** *p* < 0.05 compared to the AL group of old rats.

For the analysis of lysosomal function, vital slices that were obtained from the kidneys of AL and 35% DR rats were loaded with LysoTracker Green. We observed an increase in LysoTracker Green fluorescence intensity in kidney slices from young rats exposed to 35% DR compared to AL rats (Figure 3A). The levels of fluorescence in young AL group were the same as in old AL rats. However, the kidney slices from old rats did not demonstrate any increase in fluorescence intensity of LysoTracker Green after 35% DR (Figure 3B). Besides the estimation of average intensity, we also analyzed the coefficient of kurtosis, which measures the sharpness of the peak of dye distribution and can indirectly characterize the heterogeneity of the tubules regarding the lysosomes’ function. In young rats, the degree of kurtosis was low which could indicate that the fluorescence intensity of the tubules on the slices was distributed evenly. In old rats, the degree of kurtosis was higher, especially in the 35% DR group, indicating an increase in the heterogeneity of lysosomal activity among the tubules (Figure 3C).

### 3.3. Deterioration of Mitophagy and Mitochondrial Functions in the Kidneys of Old Rats

We assessed the function of mitochondria and the intensity of mitophagy in the kidneys of young and old rats. One of the proteins responsible for mitophagy is PTEN-induced putative kinase 1 (PINK-1). PINK-1 binds to mitochondria carrying a low membrane potential and such mitochondria become labeled by ubiquitin ligase Parkin and further eliminated in autophagolysosomes [36].

**Figure 4:**
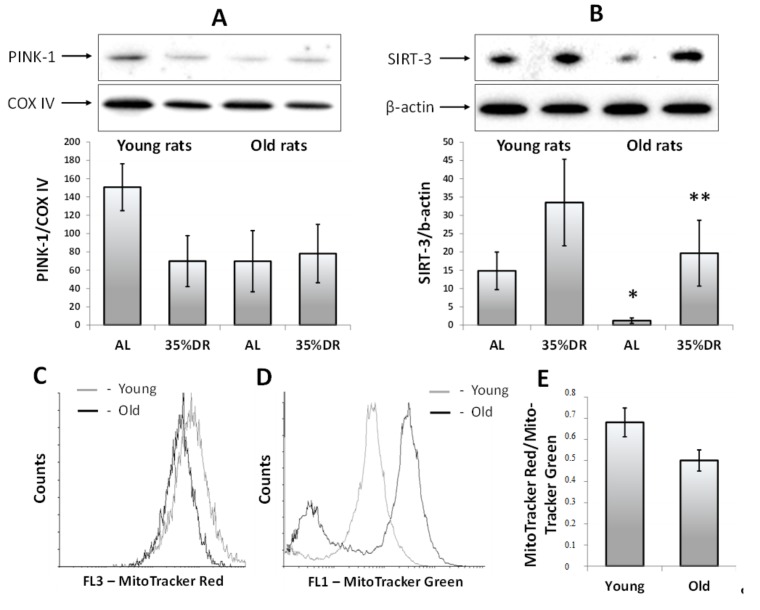
The comparison of the mitophagic activity, the level of SIRT-3, and the mitochondrial membrane potential in the kidneys of young and old rats fed AL or subjected to 35% DR. (**A**) Level of PTEN-induced kinase 1 (PINK-1) in isolated mitochondria from the kidneys of young and old rats; (**B**) Levels of SIRT-3 in kidney homogenates of young and old rats; (**C**) Flow cytometry of primary cultures of RTCs obtained from the kidneys of young and old rats, which were loaded with MitoTracker Red; (**D**) Flow cytometry of non-cultured renal tubular cells (NC-RTCs) obtained from the kidneys of young and old rats and in situ stained with MitoTracker Green; (**E**) Quantification of MitoTracker Red/MitoTracker Green ratio in NC-RTCs from the kidneys of young and old rats. * *p* < 0.05 compared to the AL group of young rats, ** *p* < 0.05 compared to the AL group of old rats.

In our experiments, we isolated mitochondria from the kidneys of rats kept on normal or restricted diets and evaluated the amount of bound PINK-1 by western blotting. We found that the level of PINK-1 dropped in the mitochondria of the young 35% DR group (Figure 4A), which might indicate the elimination of a population of poorly functioning mitochondria during 4 weeks of DR. Mitochondria in kidneys of old rats from AL group contained lower levels of PINK-1 than mitochondria from young rats. Moreover, the levels of PINK-1 did not decrease in old rats kept on 35% DR (Figure 4A), which might be a consequence of impairments in the autophagic signaling pathways in the kidneys of old rats.

We analyzed the levels of mitochondrial deacetylase SIRT-3 in the kidneys of young and old rats. In young rats, 35% DR induced an increase in the amount of SIRT-3 compared to the AL group. Old AL-rats were initially characterized by a significantly lower SIRT-3 level than in young animals. However, after 4 weeks of maintenance on 35% DR, the levels of SIRT-3 increased (Figure 4B).

We used flow cytometry to estimate the number of mitochondria and the mitochondrial potential as measured by the accumulation of MitoTracker Green and MitoTracker Red probes. The primary cultures of RTCs obtained from young animals demonstrated a higher MitoTracker Red intensity meaning that their mitochondria had higher mitochondrial potential, compared to RTCs from old animals (Figure 4C). NC-RTCs obtained directly from the kidneys of old rats and loaded with MitoTracker Green demonstrated two populations (Figure 4D), indicating the presence of cells with a small number of mitochondria. In young rats, such a segregation into two populations was not observed. Nevertheless, the MitoTracker Red/MitoTracker Green ratio revealed that NC-RTCs from the old kidneys had a lower mitochondrial membrane potential than NC-RTCs from young rats.

### 3.4. Proliferative Capacity of Primary Cultures of RTCs Obtained from Young and Old Rats

**Figure 5:**
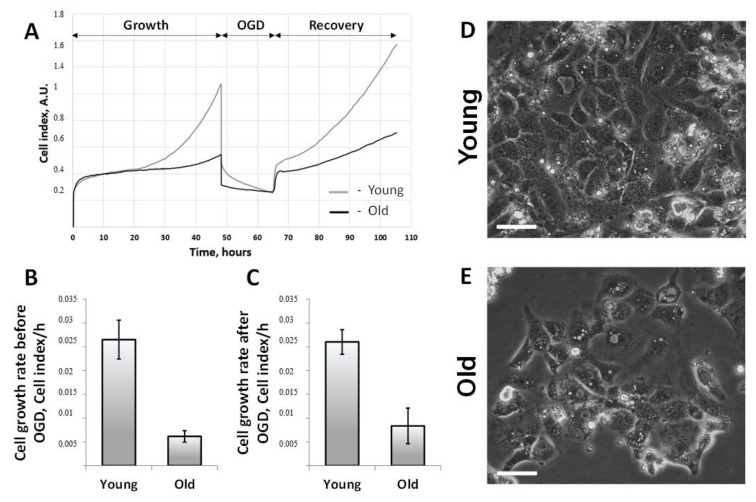
The proliferation of primary cultures of RTCs obtained from kidneys of young and old rats, and the rate of recovery after oxygen-glucose deprivation (OGD). (**A**) Kinetics of proliferation of primary cultures of RTCs evaluated by iCelligence, including growth under normoxic conditions, death during OGD, and the recovery of RTCs after the restoration of oxygen supply and complete culture medium; (**B**) Growth rate of RTCs under standard conditions; (**C**) Growth rate after OGD; (**D**,**E**) Phase-contrast microscopy images of the primary cultures of RTCs obtained from kidneys of young and old rats. The scale bar indicates 100 µm. * *p* < 0.05 compared to young rats.

We assessed the proliferative capacity of primary cultures of RTCs obtained from young and old kidneys by the real-time monitoring of the cell growth rate (Figure 5A). The analysis was performed by using iCELLigence, which has already been used for studying proliferation and the death of RTCs [32]. We found that RTCs isolated from the kidneys of young rats characterized by a more rapid growth rate exceeding the growth of old cells by more than 4 fold (Figure 5B). After 2 days in vitro, RTCs were subjected to OGD. This method simulates I/R by depriving cells of both oxygen and nutrients. During OGD, some cells died, which could be observed by the decrease of the cell index in those wells (Figure 5A). The proliferation of RTCs resumed after restoration normoxic conditions, but RTCs from old kidneys proliferated about 3 times slower after OGD than RTCs from young animals (Figure 5C).

Data from iCELLigence was visually confirmed by phase-contrast microscopy. RTC cultures were monitored every day and we verified that RTCs obtained from the kidneys of young rats proliferated more intensively and reached a higher density in a shorter time (Figure 5D,E).

### 3.5. Oxidative Stress and the Accumulation of Lipofuscin

**Figure 6:**
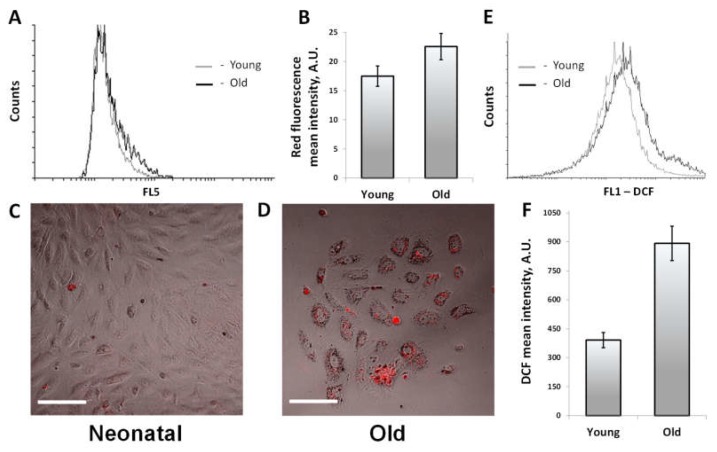
The comparison of lipofuscin content and the level of oxidative stress in cells obtained from the kidneys of young and old rats. (**A**,**B**) Flow cytometry of primary cultures of RTCs for the assessment of red autofluorescence, and its quantification for young and old rats; (**C**,**D**) Confocal images of RTCs from neonatal and old rats, and red autofluorescence emission analysis. The scale bar indicates 100 µm; (**E**,**F**) Flow cytometry of non-cultured renal tubular cells (NC-RTCs) of young and old rats and stained with DCF-H2.

It is well known that aging is associated with the accumulation of lipofuscin granules in all tissues including renal tissue [37], so we analyzed the content of lipofuscin in RTCs taken from animals of different ages. Indeed, analysis of RTCs by flow cytometry revealed that primary cell cultures isolated from old kidneys exhibited a higher red autofluorescence (Figure 6A,B), which might indicate a greater content of lipofuscin. These data were confirmed by comparison of the confocal images of RTCs. We observed that the number of lipofuscin granules was significantly higher in RTCs from old rats rather than in RTCs of neonatal cells (Figure 6C,D).

Cells from the kidneys of different ages were also compared by the levels of oxidative stress. Staining with DCF, a reactive oxygen species (ROS)-sensitive probe showed that in old kidneys cells DCF fluorescence is more than 2 times higher than in cells from young kidneys (Figure 6E,F).

## 4. Discussion

Beneficial properties of DR were discovered as early as in 1935 and since then the mechanisms of DR have been intensively studied [1]. In 2010, for the first time, a protective effect of a short-term DR was demonstrated against the deleterious action of renal I/R in young mice [27]. In our study, DR maintained for 4 weeks had a significant nephroprotective effect against renal I/R in young rats, however, it did not afford protection in the old rats.

The loss of protective effects of DR with age is gradual: in the adult, but not old, rats some beneficial effect of the DR was still observed. Probably, the manifestation of a nephroprotective effect of DR correlates with the percentage of weight loss during DR. Old rats might need longer exposure to DR to achieve a nephroprotective effect from I/R. Recent work revealed that old rats needed to be exposed to 40% DR for 8 weeks to provide a protective effect from cisplatin nephrotoxicity [15], while young animals were afforded protection after exposure to DR for 4 weeks [16]. It should be noted that in the study on mice, 100% DR for 3 days was almost as effective as 40% DR for 4 weeks [27]. However, in another study, 100% DR for 1 day did not cause any activation of the autophagic system in old mice, although in young mice even such a short period of fasting increased the amount of LC3 II in the kidney tissue [38]. We were also unable to find any nephroprotection under conditions of 100% DR both in young and old mice.

Previously, a similar loss of protective effects in old animals was reported when attempted to apply ischemic pre- and postconditioning to aged kidneys and hearts [25,26,39,40]. Such loss of effectiveness can partly be explained by the fact that during aging tissue accumulate various age-dependent alterations affecting the functioning of organs [41]. Additionally, in the kidney, structural changes ultimately expressed in the lower number of functional nephrons, degenerative changes in proximal tubules, glomerulosclerosis, as well as changes at the molecular level, are observed [42]. The aging of the kidney modulates the expression of various genes, e.g., the levels of mRNA of claudin-7, KIM-1, and metalloproteinase MMP-7, which are good markers associated with renal injury and various kidney pathologies. Prolonged DR normalizes the expression of these genes [43] and prevents the accumulation of age-dependent changes in the kidney [44]. However, even shorter periods of DR were reported to reduce age-dependent changes in the kidney [45].

Besides the disturbances in expression patterns of several genes, deterioration in the work of the autophagic system has been well documented [13]. In our experiments, we did not detect the decrease in the LC3 II/LC3 I ratio in the kidneys of old intact rats, compared to the young rats, as well as found no changes in lysosomal activity. Similar results were obtained earlier in mice [44]. This data suggests that lysosomal dysfunction rather than a decrease in autophagosome biogenesis occurs within advanced age [24]. However, we evaluated some abnormality in the biogenesis of autophagosomes in the primary culture of RTCs from old kidneys while treating cells with rapamycin and chloroquine. We subjected RTCs from young and old kidneys to incubation with rapamycin, the activator of biogenesis of autophagosomes, and chloroquine, the inhibitor of the late stages of autophagy, leading to the maximal accumulation of autophagosomes in cells. Probably, there were some changes at the biogenesis level while such treatment did not provide a significant increase in Cyto-ID fluorescence intensity in the culture of RTCs from old rats.

The pivotal role of autophagy in the normal functioning of the kidney was recently evidenced by the experiments with the knockouts of autophagy-associated genes. For instance, the deletion of *Atg5* in the podocytes led to glomerulosclerosis, proteinuria, and other abnormalities typical for the aging kidney, e.g., lipofuscin accumulation, the presence of damaged mitochondria, and oxidize or ubiquitinated proteins [46]. Genetically modified mice with tamoxifen-induced inhibition of *Atg5* in the proximal tubules demonstrated renal failure, the atrophy of kidney tissue, mitochondrial dysfunction, and an increase in oxidative stress products in the kidney [47].

It is believed that I/R stimulates autophagic cascades which provide the elimination of intracellular proteinaceous aggregates and damaged organelles formed during I/R [34]. These processes play an important role after renal I/R since the knockout of *Atg5* and *Atg7* leads to a more severe AKI [48]. Relying on the LC3 II/LC3 I ratio, we may assume that, in old rats, autophagy is activated not as effectively as it takes place in young animals. Earlier, we demonstrated impaired autophagy in response to the damaging effect of I/R in premature senescent OXYS rats [25]; that could be another argument in favor of the dysfunction of the autophagic system caused by aging. In our experiments, even a preliminary exposure to DR, which is one of the most powerful autophagy-stimulating factors, did not restore the effective work of that system in old rats after I/R. On the contrary, in young animals, DR increased the LC3 II/LC3 I ratio and the amount of lysosomes that correlated with a decrease in the severity of AKI.

The deterioration of autophagy with aging also worsens the functioning of mitophagy, which is the only mechanism for mitochondrial degradation [49]. The elimination of mitochondria with low membrane potential is very important for the cells since dysfunctional mitochondria may be the source of pathological ROS [50]. For instance, in mice with a knockout of *Atg5*, an elevated accumulation of damaged mitochondria after I/R was shown, which correlated with an increased AKI [48]. It is also suggested that the accumulation of damaged mitochondria as a result of the ineffective work of mitophagy leads to the progression of tissue aging [51]. In many organisms, aging is associated with the morphological alterations and the increased size of mitochondria [52], as well as with the accumulation of age-associated proteins [53].

In this study, we evaluated the intensity of mitophagy by the levels of PINK1 in isolated mitochondria. This protein is considered to be the marker of damaged mitochondria and the initiator of mitophagy. PINK1 carries a mitochondrial address and is delivered into mitochondria. When the mitochondrial membrane potential decreases, this protein is unable to penetrate into the mitochondria and cannot be cleaved by presenilin-associated rhomboid-like protein (PARL). Consequently, PINK1 remains located on the outer membrane of the mitochondria with a low potential, where it is marked by the ubiquitin ligase Parkin, which serves as a signal for the onset of mitophagy [54].

An increase in the levels of PINK1 is usually associated with the accumulation of damaged mitochondria in the cell. For instance, a high-calorie diet causes the rise in the levels of PINK1 whereas DR decreases the amount of this protein in mitochondria [55]. In our experiments, we observed a two-fold decrease in PINK1 levels in the mitochondrial fraction after 4 weeks of 35% DR in young rats, which shows the result of the effective elimination of low functional mitochondria. In old rats, the PINK1 levels were initially lower than in young animals, and DR did not affect the levels of PINK1. We speculate that the lower content of PINK1 in older rats may indicate a deficiency in this protein and the malfunctioning of the mechanism of mitophagy. A similar decrease of the PINK1 levels was observed in lung tissue with aging [56] and was accompanied by a pulmonary pathology. Moreover, the decrease in PINK1 activity was described in many pathological conditions, for example, in cells of patients with Parkinson’s disease [57]. It was shown that decreased PINK1 synthesis led to the fragmentation of mitochondria, decrease of the mitochondrial membrane potential [58], the development of fibrosis [59], which indicates the importance of the PINK1 protein in the normal functioning of mitochondria.

Since mitophagy is the only way to eliminate poorly functioning mitochondria, deterioration in the work of this system must inevitably lead to the accumulation of mitochondria with low membrane potentials [60]. Indeed, a large number of studies have demonstrated that mitochondria isolated from the tissues of old organisms had lower membrane potentials than mitochondria from young [52,61]. Taken together with higher levels of ROS in the tissues of old animals, it is assumed that age impacts either on the functioning of mitochondria or on the process of their quality control [50]. Our findings fit this assumption as we showed a lower mitochondrial membrane potential in kidney cells isolated from old rats, as well as a higher heterogeneity in mitochondria of cells obtained from old rats’ kidneys revealed by flow cytometry. A similar heterogeneity of mitochondrial membrane potential was observed in hepatocytes from old rats [62]. Earlier, the phenomenon of the augmentation of the mitochondrial membrane potential heterogeneity in the kidney tissue after I/R was reported [63]. We also revealed an increase in LysoTracker Green distribution heterogeneity in the tubules of old rats. Probably, the increase in heterogeneity is a process that accompanies the aging of tissues.

Another important factor reflecting the state of mitochondria is the activity of deacetylase SIRT-3. This NAD^+^-dependent deacetylase is localized predominantly in mitochondria and is considered to be a mitochondrial stress sensor that can modulate the activity of several mitochondrial proteins involved in metabolism and oxidative stress regulatory pathways [64]. SIRT-3 is suggested to be one of the mediators of the protective effect of DR [65]. In this study, DR increased the levels of SIRT-3 more than 2 times in the kidneys of young rats. In kidneys of old rats, the levels of SIRT-3 were significantly lower than in young, while DR significantly increased the content of this deacetylase. An age-associated drop of mitochondrial SIRT-3 was previously reported for some tissues of old people and old animals [66,67]. Contrarily, some variants of the *SIRT-3* gene were associated with longevity in humans [68], proving the key role of this deacetylase in the aging process.

Recent studies have demonstrated that increased levels of SIRT-3 protect the kidney from cisplatin nephrotoxicity [69], whereas the knockout of *SIRT-3* led to more severe damage after myocardial infarction [70]. The levels of SIRT-3 depended on physical activity and diet, e.g., the levels of the deacetylase rose in muscles after exercise [71] and in cells exposed to decreased glucose concentration in culture medium [72]. In addition, the knockout of *SIRT-3* in the kidneys of mice caused fibrosis, increased in the TGF-β1 level and the hyperacetylation of the kinase of glycogen synthase 3β (GSK3β) that adversely affected its phosphorylating activity [73]. Similarly, another deacetylase, SIRT-1, was detected to be lower in old animals, while 12-month-long DR increased the levels of this enzyme [44].

Our experiments with cell cultures showed that primary RTCs obtained from kidneys of old rats proliferated 4 times slower compared to cells from the kidneys of young rats. Such a dependence of proliferation rate on the age of the donor was demonstrated for various cells. For instance, hematopoietic stem cells derived from neonatal rats proliferated much better, and, unlike stem cells derived from adult rats, they were able to exhibit protective properties under myocardial I/R [74]. Although cardiomyocytes obtained from adult animals are thought to represent processes in the tissue better than neonatal cardiomyocytes, they proliferate much more slowly in culture [75].

Furthermore, primary kidney cells from old rats not only proliferated worse than those of young rats, but we also observed their less pronounced recovering ability after OGD. We believe that this impairment could be explained by deterioration in the functioning of the autophagy system, which could lead to the accumulation of lipofuscin, as well as to more pronounced oxidative stress.

## 5. Conclusions

In this study, we showed that a short-term 35% DR for 4 weeks has a significant nephroprotective effect against renal I/R in young rats, but DR does not provide such a beneficial effect in old rats. The lack of effectiveness in old animals can be explained by the deterioration in the work of both autophagy and mitophagy. Impairment in these recycling mechanisms results in accumulation of poorly functioning mitochondria, lipofuscin granules, and increased levels of oxidative stress. All these processes affect the normal functioning of the kidney and lead to a decrease in the ischemic tolerance of renal tissue. Molecular and structural alterations occurring in aging renal tissue make the manifestation of beneficial effects of DR in aged kidneys not as pronounced. Thus, an important problem is to develop approaches that would be helpful in the therapy of elderly patients.
